# Network analysis methods for studying microbial communities: A mini review

**DOI:** 10.1016/j.csbj.2021.05.001

**Published:** 2021-05-04

**Authors:** Monica Steffi Matchado, Michael Lauber, Sandra Reitmeier, Tim Kacprowski, Jan Baumbach, Dirk Haller, Markus List

**Affiliations:** a Chair of Experimental Bioinformatics, Technical University of Munich, 85354 Freising, Germany; b ZIEL - Institute for Food & Health, Technical University of Munich, 85354 Freising, Germany; c Chair of Nutrition and Immunology, Technical University of Munich, 85354 Freising, Germany; d Division Data Science in Biomedicine, Peter L. Reichertz Institute for Medical Informatics, TU Braunschweig and Hannover Medical School, 38106 Brunswick, Germany; e Institute of Mathematics and Computer Science, University of Southern Denmark, 5230 Odense, Denmark; f Chair of Computational Systems Biology, University of Hamburg, 22607 Hamburg, Germany; g Braunschweig‌ ‌Integrated‌ ‌Centre‌ ‌of‌ ‌Systems‌ ‌Biology‌ ‌(BRICS),‌‌ 38106 Brunswick,‌ ‌Germany

**Keywords:** Microbial co-occurrence networks, Microbial interactions, Network analysis, Trans-kingdom interactions

## Abstract

Microorganisms including bacteria, fungi, viruses, protists and archaea live as communities in complex and contiguous environments. They engage in numerous inter- and intra- kingdom interactions which can be inferred from microbiome profiling data. In particular, network-based approaches have proven helpful in deciphering complex microbial interaction patterns. Here we give an overview of state-of-the-art methods to infer intra-kingdom interactions ranging from simple correlation- to complex conditional dependence-based methods. We highlight common biases encountered in microbial profiles and discuss mitigation strategies employed by different tools and their trade-off with increased computational complexity. Finally, we discuss current limitations that motivate further method development to infer inter-kingdom interactions and to robustly and comprehensively characterize microbial environments in the future.

## 1 Introduction

**Fig. 1:**
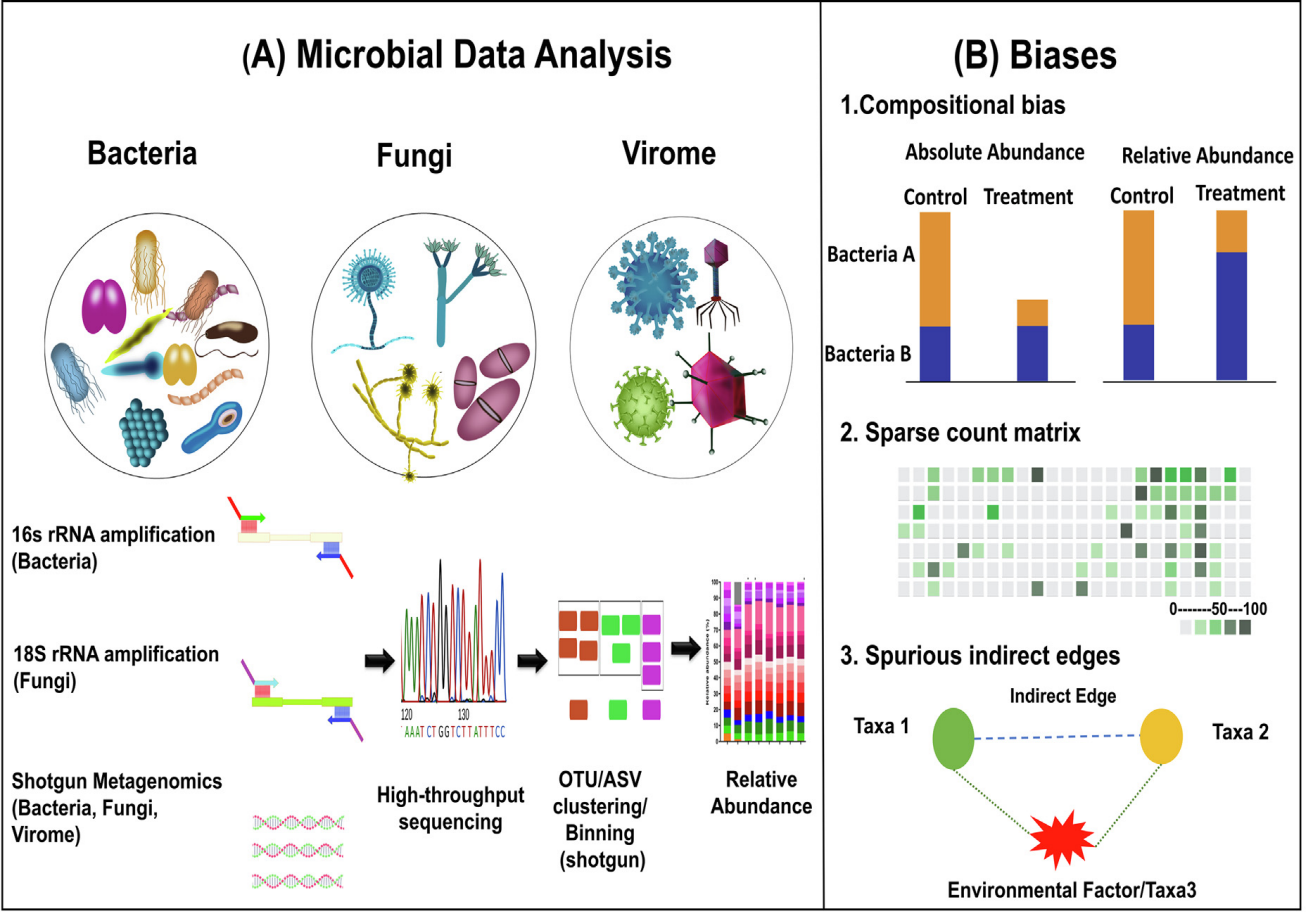
(A) Schematic overview on taxonomic profiling of bacteria, fungi and the virome. (B) Illustrates three important biases: compositionality, sparsity and spurious correlations in microbial co-occurrence network analysis.

The human body acts as a host for complex microbial communities consisting of bacteria, protozoa, archaea, viruses and fungi [1]. Next-generation sequencing techniques proved very effective for characterizing microbial communities by sequencing suitable molecular targets such as 16S ribosomal RNA gene amplicons for bacteria, internal transcribed spacer regions of ribosomal RNA genes for fungi and shotgun metagenomics for viruses (Fig. 1A). Since these organisms share the same host, they are in constant competition, where some organisms develop symbiotic relationships in which they cooperate or synergize with each other for gaining a fitness advantage that may or may not benefit the host organism [2], [3]. Thus far, microbiome research has mostly focused on interactions between the host and its microbiome, mostly on the level of bacteria. However, *trans*-kingdom interactions between bacteria, fungi and viruses, as well as their joint effect on the host, have only recently been studied [4].

Network-based analytical approaches have proven useful to study systems with complex interactions and represent a powerful tool in systems biology to infer gene-regulatory and other complex networks [5], [6], [7]. The complex interactions between thousands of individual species across kingdoms as found, for instance, in the human gut microbiome, suggests that such network analysis methods are also useful in the microbiome field. In this review, we first highlight network analysis methods that have already been used successfully for inferring community structures from bacterial abundances. Next, we focus on recently developed and repurposed methods that have been used for *trans*-kingdom analysis. Finally, we will discuss why more concerted efforts in network method development are necessary to address the unique aspects of microbial data.

## 2 Network methods for microbial communities

Until now, bacterial co-occurrence patterns were studied extensively while fungal or viral interactions have received less attention [8], [9]. Systems and network biology approaches have been used to decipher microbial co-occurrence patterns and range from correlation methods to complex graph-based models. A recent study investigating the earth microbial co-occurrence network identified connections across fourteen different environments, including plants, animals, water and soil [10]. The earth microbial co-occurrence network thus highlights the importance of studying microbial interactions across microbial niches using suitable tools.

Decoding complex microbial co-occurrence relationships is associated with three main challenges. Firstly, microbiome data are compositional [11]; i.e. microbial counts represent proportions instead of absolute abundances. Secondly, sparsity in the dataset can lead to false associations of microorganisms. A zero indicates either the absence of a microorganism, or an insufficient sequencing depth. Thirdly, it is challenging to differentiate between direct and indirect associations, in particular if these are related to environmental factors (Fig. 1B).

**Fig. 2:**
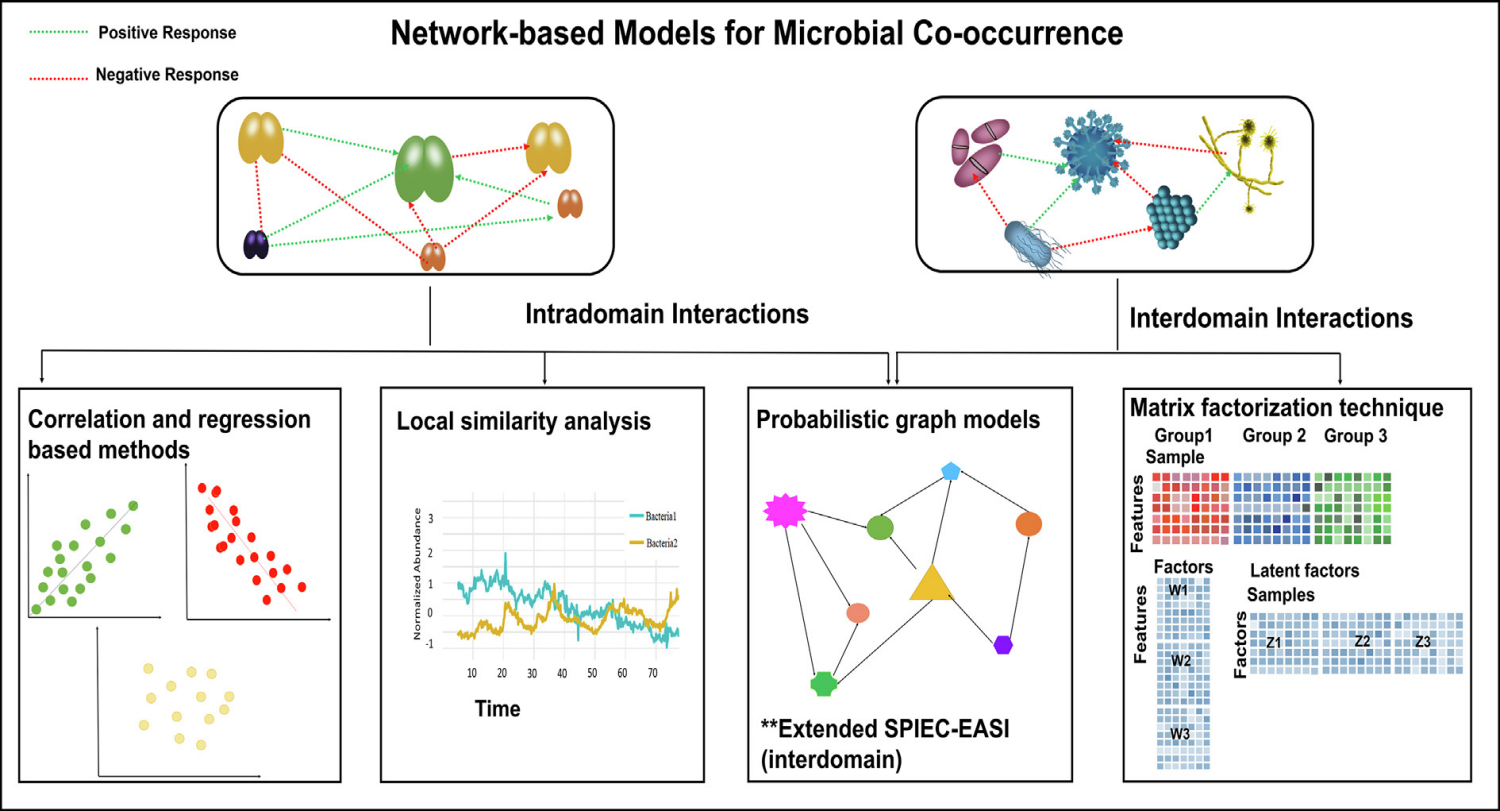
Overview of network approaches for microbial intra- and inter-kingdom interactions.

Correlation-based techniques, including Pearson or Spearman correlation, are among the most popular methods for studying microbial interactions in human gut [12], oral [13] and soil [14] microbiomes. Weiss et al. [15] evaluated the strengths and weaknesses of eight different correlation methods and provided recommendations based on the nature of the data and identified sparsity as a key issue not sufficiently addressed by these approaches. Correlation analysis often results in artefactual and spurious associations between low-abundant microbial members in a community as it fails to account for compositionality [11]. As Lovell et al. [16], [17] showed, correlation-based methods are not subcompositionally coherent such that, for instance, depleting rare taxa is expected to change the outcome of correlation analysis. To overcome this issue, compositional data analysis can be employed. Various proportionality measures [16], [17] have been proposed some of which are implemented in the R package propr [18] and can be used for network construction. A frequently used method to account for compositionality is centered log ratio transformation (CLR) [19], [20], where the geometric mean of the sample vector is used as the reference. CLR transformation maps the relative counts from simplex into Euclidean space and hence makes these data compatible with linear analysis methods. Apart from these classical approaches, more complex methods have been proposed based on probabilistic graphical, Gaussian graphical and complex multiple regression models to construct microbial interaction networks [6], [21]. Most methods take compositionality into account either by performing CLR transformation as a pre-processing step or by using a Dirichlet multinomial model to directly account for compositionality. Existing methods differ with respect to sensitivity, specificity and computational complexity and can be grouped into four different categories (Fig. 2). In the following, we describe the underlying concepts of tools (Table 1 and Supplemental Material) that have been successfully applied in the analysis of microbial data in humans [22] as well as other environments [23].

**Table 1:** Overview of microbial co-occurrence network methods.

Tools	Principle/Models	Advantages	Limitation	Applications
**Correlation based Methods**				
SparCC (2012) python r-sparcc	• Pearson correlations from log-transformed abundance • Bayesian approach to differentiate true fractions from the observed counts and to handle sparsity • Log-ratio transformed abundance/count matrix	• Handles compositionality bias and sparsity	• High computational complexity due to the iterative approximation approach • Nonlinear relationships cannot be detected	• Interaction between gut fungi microbiome of the Human Microbiome Project healthy cohort [105] and other studies including identifying biomarkers in of diet and lifestyle [28], interaction between Mucosal microbiome in gastric carcinogenesis [106] etc
CCLasso (2015) R package	• Latent variable model with l1-norm shrinkage method • simple pseudo count implementation • Log-ratio transformed abundance/count matrix	• Faster than SparCC • Handles Compositionality bias	• Nonlinear relationships cannot be detected • Study only pairwise correlations between microbiomes [107]	• It was used to capture the interaction between Marine phototrophs and archaea [108]
REBACCA (2015)	• Linear system using log ratios between pairs of compositions with l1-norm shrinkage method	• Obtain higher accuracy when a sparse condition is satisfied • Controls the false positives • Suitable for large sample size	• Nonlinear relationships cannot be detected • Asymptotic performance with large sample size	• Positive correlation between *S. amnii, BVAB1, Prevotella* cluster 2 and TM7-H1 which are associated with Preterm birth. It also helps to report the first report of an association of TM7-H1 with PTB [109]
CoNet (2016) Cytoscape Command line tool	• Five similarity measures: Bray and Curtis, Kullback–Leibler dissimilarity measures, Pearson and Spearman correlation, and mutual information • Compendium of generalized boosted linear models	• Able to build bipartite network	• Does not address compositionality bias • Study only pairwise correlations between microbiomes [107]	• Interaction studies of ecological systems like ranging from plant [110], [111], soil [112] to human microbiome. • Identification of autism spectrum disorder-enriched *F. prausnitzii, B. uniformis*, and *B. vulgatus* [113]
Meta-Network (2019)	• Hybrid method with Pearson Correlation and graph-based method FS-Weight method to study indirect relationships • Nonlinear associations using PCA-PMI method • MCODE cluster algorithm to detect clusters and hubs	• indirect correlation and non-linear correlations can be identified • Outperforms the Spearman and Pearson Correlation	• Does not address compositionality bias	• Identification of hidden relationship between *Syntrophomonas* and *Methanogens* which plays a vital role in transferring the short-chain fatty acid into methane and energy [36].
Correlation-Centric Network (2020) Command line tool	• Edge-centric Network • Pearson correlation coefficient for network construction • Isomorphism mapping for deriving Correlation-Centric Network from species–species co-occurrence networks (SCNs)	• Correlations of the edge distribution can be studied • Outperforms the SCNs	• Does not address compositionality • CCN a new perspective in microbiome network derived from host diet during the seasonal variations. Identified	• Identification of biomarkers in gene-co-expression and personalized characterization of diseases [114] and also in time-series human gut microbiome data [89]
MENAP (2012) online tool	• Random Matrix Theory (RMT)- based molecular ecological network analysis	• Threshold to construct network is automatically determined • Robust to noise	• Does not address the issues of network sparsity and compositional bias	• Detection of highly connected cluster of *Fusobacterium* in oral bacterial dysbiosis and oral squamous cell carcinoma (OSCC) [115] • Study of soil microbial structures [116], [117], [118]
**Conditional Dependence/graphical Models**				
gCoda (2017) R package	• Logistic normal distribution to overcome compositionality-bias • Majorization-Minimization algorithm • Maximum likelihood with l1 penalty to deal with dimensionality	• Requires less computation time than SPIEC-EASI • Efficient for compositional data • More stable and accurate compared to SPIEC-EASI.	• Non-convexity of the likelihood function [119] • Lack in identifying the hub/Key species [120] • Lack in consistency of the estimators [121]	• Not available
MDiNE (2019) R package	• Dirichlet-multinomial logistic-normal distribution to address the compositional nature • Markov Chain Monte Carlo (MCMC) methods to define logistic multinomial normal model	• Differential networks based on precision matrix estimation for binary sample condition • Zero handling without resorting to the addition of a pseudo-count • Handles Compositionality	• Running time is high • Supports only single binary covariate to construct the networks • Dirichlet-multinomial logistic-normal distribution model cannot capture positive and negative covariances [56]	• Identification of new biomarkers such as Enterobacteriacea, more abundant in Crohn’s samples and Lachnospiraceae to be less [60]
MixMPLN (2019) R package	• Mixture of K Multivariate Poisson Log-Normal distributions • Minorization–maximization principle • ℓ1-penalty model to solve the sparse networks	• Capturing multiple networks from the same count matrix • Handles Compositionality	• Runtime comparison and computational complexity are not well-addressed.	• Able to reproduce and identify the changes between infants gut microbiome and older children and adults [56]
NetComi (2020) R package	• Integrates extensive list of methods that take into account the special characteristics of amplicon data: SparCC, SPIEC-EASI, proportionality, SPRING • unique feature: Differential network analysis	• Ability to study differential networks • Easy-to-use	• Model networks from a single domain of life	• Not available
Environmentally-Driven Edge Detection (2020)	• Sign Pattern, Overlap, Interaction Information, Data Processing Inequality to remove the environmentally-driven (indirect) associations	• Ability to identify the environmentally-driven (indirect) associations (edges) from the network	• Currently ENDED supports only any closed triplet i.e (fully connected)	• Not available
Mint (2015) R package	• Poisson-multivariate normal hierarchical model with ℓ1-penalty model to capture direct interactions	• Controls for confounding predictors to remove indirect interactions	• Does not account for the compositional nature of microbiome data • Unable to detect latent factors	• Not available
mLDM (2016) R package	• Hierarchical Bayesian model with sparsity constraints	• Handles compositional bias • Able to detect direct associations and remove indirect associations • Microbial absolute abundance can be estimated	• Lacks scalability and efficiency, high computational power • Hierarchical Bayesian model consume most of the training time. • Unable to detect latent factors	• Not available
HARMONIES (2020) R package webtool	• A hybrid approach using Zero-inflated negative binomial distribution and Dirichlet process • Gaussian graphical model to deal with sparse network	• Handle overdispersion and high number of zero counts	• Small sample size affects the performance	• Discovered a unique subnetwork of Fusobacterium, Peptostreptococcus, and Parvimonas in healthy patients compared to Colorectal cancer patients [122]
SPIEC-EASI (2015) R package	• CLR transformation of the input • Selection of two approaches: Glasso or Neighborhood Selection	• Handles compositionally • Avoids detection of transitive correlations	• Graphs with large hub node are more difficult to recover • Cannot handles co-variates	• Interaction studies of various ecological systems like plants [23], murine [123] and human. • Study the interactions of Viral Populations to identify the Age-Dependent patterns in human gut [124]
Hubs weighted graphical lasso (2020)	• Weighted lasso approach with special row/column sum weights to penalize hubs	• Includes structural information of the network to correctly identify hub edges		• Not available
FlashWeave (2019)	• Local-to-global learning framework	• Adjusts for latent variable • Less runtime • Good performance on heterogenous datasets	• Quality drop when applied to homogeneous data with small sample number	• Understanding the interaction between Core Microbiome of ascidian, a marine invertebrate chordates [125]
COZINE R package (2020)	• CLR transformation only on non-zero count values • Multivariate Gaussian Hurdle model • Group-lasso penalty to obtain sparse estimates	• Handles compositional bias and zero inflation • High accuracy		• Not available
**Network-based methods for *trans*-kingdom analysis**				
SPIEC-EASI Extension (2018) R package	• Central Log Ration Transformation of the input • Selection of two approaches: Glasso or Neighborhood Selection	• Handles compositionally • Avoids detection of transitive correlations	• Graphs with large hub node are more difficult to recover	• Identification of associations between fungi and bacteria and also elucidated the importance of including cross-biom interactions in microbiome data analysis [90]
Multi-Omics Factor Analysis R package (2018)	• Normalized data matrices from one or more data modalities • Bayesian Group Factor Analysis framework • Automatic Relevance Determination	• Integrates multiple data modalities and sample groups and finds drivers of variation	• Assumes linear or moderate non-linear relationships • Assumes independence between features in prior distribution	• Identification of complex interaction between *trans*-kingdom during antibiotic perturbation [93]
DIABLO R package (2019)	• Singular value decomposition with ℓ1-penalized selection of correlated variables from several omic data sets	• Finds correlated features which possess a discriminative ability	• Assumes linear relationship between features from different omics data sets	• Interaction study between bacterial taxa, metabolites and physiological traits in the study of haem-induced lipoperoxidation on mucosal and luminal gut homeostasis [126]

## 3 Correlation based methods

Many correlation-based methods employ variants of Pearson or Spearman correlation to obtain an estimate of microbial interaction between pairs of taxa [24], [25]. However, these measures do not account for compositionality, where, for instance, an increase in absolute abundance of just a single taxon is followed by a decrease in relative abundances of all other taxa even if their absolute abundance does not change (Fig. 1B) [11]. This can be mitigated by ratio transformation of the data. Ratio transformations ensure that the ratios between two features are the same whether the data are absolute counts or proportions. Taking the logarithm of these counts makes the data further symmetric and linearly related [19]. The resulting correlation coefficients are thus compositionally coherent, i.e. the log ratio of two taxa is completely independent of other taxa. Sparse Correlations for Compositional data (SparCC) [26] is a popular method employing this strategy with applications ranging from human gut microbiome studies [27], [28], [29] to environmental studies. SparCC is based on an iterative approximation approach and uses log-ratio transformed data to infer the correlations between the components. Under the assumption that the underlying networks are large-scale and sparse. SparCC was shown to be better suited to avoid spurious correlations compared to direct Pearson correlations [15] at the cost of higher computational complexity [30]. Another strategy that was proposed to improve the robustness of correlation coefficients is bootstrapping as implemented in CoNet [24]. CoNet further employs similarity (Steinhaus, distance correlation) and dissimilarity measures (Euclidean, Jensen-Shannon, Kullback Leibler, Bray Curtis) as alternatives to correlation coefficients. Another challenge in correlation-based networks is the choice of a suitable correlation cut-off which controls the sparsity of the resulting network. While the choice for the cut-off is often left to the user, the Molecular Ecological Network Analysis Pipeline (MENAP) [25] offers an automated selection of the optimal correlation threshold via a random matrix theory-based method [31] to simulate a random background.

## 4 Regularized linear regression

An alternative to correlation methods is to build linear regression models in which the abundance of each taxon is modelled as a response variable using the abundance of all other taxa as explanatory variables. Here, the coefficient of each taxon serves as a linear measure for the interaction strength of two taxa. However, due to the large number of features, such models are generally prone to overfitting. A common strategy to mitigate this issue is to introduce a penalty term, yielding regularized regression models. Here, the ℓ1-penalty, also known as lasso, is typically used to drive the coefficients of taxa with negligible contribution to zero, thus increasing the sparsity of the solution. For instance, Correlation inference for compositional data through Lasso (CCLasso) [32] and Regularized Estimation of the BAsis Covariance based on Compositional dAta (REBACCA) [33] use this strategy to build a regularized correlation network of microbiome data. CCLasso also adapts CLR transformation to address compositionality, while REBACCA models the log basis covariance structure to directly account for compositionality. While CCLasso and REBACCA perform similar to SparCC in terms of reproducibility and consistency, regularization appears to be beneficial for avoiding the detection of spurious relations [32]. In addition to the existing lasso methods, Bates and Tibshirani [34] proposed a new ℓ1-penalized regression model based on all-pairs log-ratios for sparse estimation. The all-pairs log-ratio model overcomes compositionality, increases accuracy and leads to improved interpretability. Further, Lu et al. [35] introduced ℓ1-penalized generalized linear regression models (GLMs) with linear constraints that achieve sub-compositional coherence.

## 5 Association rule mining

Instead of regularization, Meta-Network [36] uses advanced association rule mining [37] to detect intricate (i.e. including indirect and non-linear) correlations. To this end, Meta-Network first generates presence-absence indicator matrices for each sample. Subsequently, the co-occurrence frequencies of taxa pairs are computed yielding a co-occurrence probability matrix. This matrix is then used to construct a network with a co-occurrence probability of e.g. 80%. (default threshold in Meta-Network). Following this loose definition, Meta-Network uses the graph-based Functional Similarity Weight (FS-Weight) [38] algorithm to detect indirect relationships and the PCA-PMI [39] method (Path Consistency Algorithm) to infer non-linear associations. These two methods (FS-Weight and PCA-PMI) are able to independently capture many of the same nodes and edges which, according to the authors, indicates that they both can depict the complex nature of the microbial relationships.

## 6 Conditional dependence and graphical methods

Correlation based methods typically fail to differentiate between direct and indirect associations. To account for this, a plethora of methods have been developed to model conditional dependence which usually have a higher computational complexity and run-time than correlation-based methods. Partial correlation [40] and related approaches are used here to distinguish between direct and indirect interactions, resulting in an undirected weighted graph where the edges imply the conditional dependency between two taxa.

Most of these methods can also account for confounders such as biological covariates and technical biases such as sequencing depth. For instance, Mint (MicrobialInteraction) employs a Poisson-multivariate normal hierarchical model to identify direct microbial interactions while controlling for user-provided confounders at the multivariate normal layer using an ℓ1-penalized precision matrix [41].

Two different strategies are implemented in SPIEC-EASI (SParse InversE Covariance Estimation for Ecological Association Inference) [42] after applying a CLR transformation to the data to address compositionality. The first method generates a graphical network by estimating a sparse inverse covariance matrix (sparse graphical model inference with Glasso) [43] and the second method employs the Meinshausen-Bühlman method, a node wise regression model [44], [45]. SPIEC-EASI infers the appropriate amount of sparsity of a network by using the Stability Approach to Regularization Selection [46].

A number of methods were inspired by SPIEC-EASI and mostly differ in the models they employ to infer conditional independence. For instance, gCoda [47] also performs CLR transformation on the relative abundance but then uses a logistic normal distribution to model the counts and a maximum likelihood model with ℓ1-penalty to deal with sparsity. According to the authors, gCoda surpasses SPIEC-EASI in terms of stability, accuracy and runtime.

Another method, metagenomic Lognormal-Dirichlet-Multinomial (mLDM) [48] affords a more complex hierarchical Bayesian model with three layers. First, mLDM models the count matrix by using a multinomial distribution. Second, a Dirichlet distribution is used to model the multinomial probabilities and, finally, mLDM utilizes a multivariate log-normal distribution to model the absolute microbial abundance [48]. The authors could show that mLDM performed favourably compared to Pearson and Spearman correlation, SparCC, CCLasso, CCREPE, glasso and SPIEC-EASI in terms of finding true taxa-taxa and environmental factors and taxa associations. However, this multi-layer approach leads to high computational complexity and limits both scalability and interpretability [47].

Hybrid Approach foR MicrobiOme Network Inferences via Exploiting Sparsity (HARMONIES) [49] employs the zero-inflated negative binomial distribution (ZINB) and a Dirichlet prior to deal with overdispersion and the large number of zero counts. HARMONIES then uses a graphical lasso approach to infer interactions with favourable results compared to SPIEC-EASI (using both Glasso and the Meinhausen-Bühlmann method), and CClasso on synthetic data, in particular when additional zeros were added.

Most of the methods which try to solve zero-inflation, introduce pseudo counts before log transformation. Ha et al. [50] discussed that introducing pseudo counts may have a huge impact on downstream analysis and also may lead to spurious associations i.e. neglecting the fact that some taxa are completely absent in the data. To overcome this, Ha et al. [50] proposed a new COmpositional Zero-Inflated Network Estimation (COZINE) model where they generate a binary incidence matrix and a compositional abundance matrix in which CLR is applied only to the non-zero count data. A Multivariate Gaussian Hurdle model [51] with group-lasso penalty is then fitted into the combined form of binary and continuous matrix to infer the three types of interactions: binary-binary, binary-continuous and continuous-continuous relations. By doing so, COZINE tries to accommodate both compositionality and zero inflation.

## 7 Addressing network topology bias

Topological features of the network such as the node degree may introduce a bias in statistical inference, where highly connected taxa (hubs) have disproportionate influence. The vast majority of methods do not consider topological network features since they implicitly assume conditional independence [52]. Recently, McGillivray [53] proposed a weighted graphical lasso approach that incorporates row/column sums as weights to penalize hub edges. The method performed significantly better compared to competing methods including graphical lasso, adaptive graphical lasso or hubs graphical lasso.

## 8 Methods scaling to large-scale data

A general issue with probabilistic graphical model approaches is their lack of computational scalability. FlashWeave [54] mitigates this issue by using a modified version of the semi-interleaved HITON-PC algorithm [55], a causal inference algorithm which infers for each taxon its Markov blanket. Given its Markov blanket, a taxon is conditionally independent of every other taxon in the graph. The algorithm starts by labelling for each taxon T significantly associated taxa, based either on Pearson correlation or mutual information, as candidate neighbours. Then only taxa which are conditional dependent from T given all combinations of other neighbour taxa are kept. Individual neighbourhoods are subsequently connected. To achieve scalability FlashWeave uses a set of heuristics and optionally incorporates metadata to disentangle direct microbial associations from confounding factors introduced in cross study analyses.

## 9 Multi-view networks

Most of the network methods assume that the sample-taxa matrix is associated with a single network, i.e. there is only one network topology with a set of edge weights. However, a sample-taxa matrix may be derived from a larger number of biological samples where taxa may be associated with more than one network topology. Especially the human gut microbiome is associated with various factors including diet, age and health and, hence, the associated microbial network may vary according to the influence of these factors. Tavakoli et al. [56] used a mixture model based on the Multivariate Poisson Log-Normal (MPLN) distribution [57] to build K microbial networks from a sample-taxa matrix associated with K underlying distributions. Similar to Mint [41], MixMPLN uses a Poisson-multivariate normal hierarchical model to capture the direct microbial interactions. However, different to Mint, MixMPLN constructs one network for each confounder and infers the parameters of the distributions using a maximum likelihood framework based on the Minorization–Maximization (MM) algorithm [58]. In addition, MixMPLN also uses the ℓ1-penalty to regularize the sparsity of the networks. The authors extended this idea also to other algorithms such as MixGGM and MixMCMC [59]. MixMCMC utilizes Markov Chain Monte Carlo model to evaluate the latent parameters in the MPLN mixture framework. MixGGM utilizes Gaussian distributions on CLR transformed- abundance matrix to overcome compositionality bias.

## 10 Differential network analysis

While most methods construct a single co-occurrence network irrespective of study conditions such as disease, treatment or control, it is in many cases the differences between such conditions that are of greatest interest. To account for this, a few methods have been developed for differential network analysis.

Microbiome Differential Network Estimation (MDINE) [60] generates differential networks to show how microbial relationships vary between two conditions based on an estimation of the precision matrix. MDiNE addresses compositionality by utilizing a Dirichlet-multinomial logistic-normal distribution model [61], [62]. Apart from handling compositionality, multinomial logistic models are also suited to handle the large number of zeros in microbial abundances without reverting to pseudo counts. In contrast to MDiNE, NetCoMi [63] utilizes permutation tests to evaluate the significantly different taxa between the groups. More specifically, NetCoMi performs differential association analysis using Fisher’s z-test [64], a non-parametric resampling procedure [65] and the discordant method [66] to build differential networks that are limited to differentially associated taxa.

## 11 Inferring interaction types

In ecological networks, microbial interactions are shown as directed edges from a source to a target species, where different types of interactions can be modelled, e.g. competition, mutualisms or parasitism [67]. In contrast, microbial association networks are typically undirected and not all interactions represent true ecological relationships. EnDED [68] aims to differentiate direct and indirect associations based on environmental factors which may affect the dynamics of the ecosystem, such as temperature, turbidity, salinity and nutrients. It employs four different approaches, such as Sign Pattern [69], Overlap [68], Interaction Information [69], [70], and Data Processing Inequality [71], [72] to identify indirect (environmentally-driven) edges. It classifies an edge as indirect due to environment factor, only if all four methods classify it as indirect.

Alternatively, Lotka–Volterra models are commonly used to predict different types of interactions. While classical Lotka-Volterra models are used to predict predator–prey (competition) interaction between two species, the generalized Lotka–Volterra (gLV) [73], [74] uses a logistic model to simulate the growth of microbes and to infer whether an interaction of two species is competitive, amensalistic or predator–prey [75]. However, since gLV-based models estimate dynamics with respect to absolute abundance, a new nonlinear dynamical system called compositionally aware Lotka-Volterra method (cLV) [76] was developed. cLV predicts microbial dynamics in-terms of ratio of relative abundance between taxa. Joseph TA et al. [76] compared the performance of cLV against gLV using simulated and real datasets and showed that cLV forecasts microbial interactions more accurately compared to gLV.

## 12 Studying microbiome time-series dynamics

Microbiomes tend to change their compositions in response to perturbations of their environment. Time-series analysis aims to study dynamic interaction changes in microbial compositions to reveal contemporaneous patterns and factors which are responsible for changes in the community behaviour. Faust et al. [77] discussed different network inference techniques to investigate temporal changes in microbiome studies including local similarity analysis (LSA) [78], Time-decay analysis [79], Augmented Dickey Fuller test [80], Cross correlation [80], Time-varying network inference [81], Hurst exponent [82], Bistability analysis [83], as well as Extended LSA (eLSA) [84], which offers support for replicates. Among these techniques, LSA is the most commonly used method to study dynamic changes. It utilizes dynamic programming to detect changes between the time series and to identify associations based on a similarity score. Alternatively, Dynamic Bayesian networks and temporal event networks can be used to study the temporal changes in microbial data. Dynamic Bayesian networks have been successfully used to study the changes in microbial compositions of the infant gut microbiome [85], other longitudinal microbiome data including vaginal and oral cavity microbiome [86].

The majority of the microbial network tools emphasize nodes (representing taxa at different taxonomic levels) but only limited attention is given to the edges capturing their associations [87], [88]. Although these may delineate important dynamic changes of microbial co-occurrence. Correlation-Centric Network (CCN) [89] transforms the node into an edge graph, where nodes represent the co-occurrence of two taxa while edges represent one of the two co-occurring taxa, respectively. This correlation-centric network representation is hence suited to capture dynamic changes in the microbial environment [89].

## 13 Network-based methods for *trans*-kingdom analysis

During the last years, the continuously dropping costs for high throughput sequencing technologies allowed scientists to go beyond the mere characterization of the bacterial part of the microbiome and to investigate the role of viruses and fungi within the microbial community. Methods using the information of several data modalities concurrently are thus sought to deliver insights into the relation between taxa from different kingdoms and to gain a more comprehensive understanding of the microbial system. Since such multi-modal data is still relatively scarce in the microbiome community, only few methods have been developed and applied for this purpose. For instance, Tripton et al. [90] adapted the SPIEC-EASI method for *trans*-kingdom analysis by concatenating two or more data sets which were independently CLR-transformed. The combined data is then used to estimate a sparse inverse covariance matrix which can be interpreted as an intra- and cross-domain interaction network. Applying their method on data from lung and skin bacteria as well as from the fungal microbiome, the authors showed that cross-kingdom networks had a higher overall connectivity and that the modularity was reduced compared to the single-domain networks.

However, the SPIEC-EASI extension does not offer insights into underlying factors driving the variation across samples or different groups of samples. To achieve this, Argelaguet et al. [91] proposed a method called Multi-Omics Factor Analysis (MOFA) which uses group factor analysis [92] to provide an integrative analysis of a set of samples with measurements from different data modalities, making it an attractive tool for *trans*-kingdom analysis as demonstrated by Haak et al. [93].

Similarly, Data Integration Analysis for Biomarker discovery using Latent cOmponents (DIABLO) [94] is a multi-omics integration tool based on partial least squares (PLS) regression, a technique to reduce the number of predictors by finding a small set of uncorrelated variables which are then used to perform least squares regression and was successfully applied to multi omics data set consisting of microbiome, metabolome, proteome und mRNA measurements, where it revealed discriminatory biomarkers for fibromyalgia patients [95]. However, in contrast to MOFA, DIABLO supports only continuous variables and assumes a linear relationship between the selected variables which may not be given, in particular in such complex scenarios.

## 14 Discussion

Microorganisms have built complex and robust ecosystems in various environments ranging from soil or sea water to various organs of the human body. Understanding the nature of microbial co-occurrence and correlation patterns within and between kingdoms may thus provide insights into the robustness of ecological systems and offer insights into complex human diseases such as inflammatory bowel disease, which is known to be influenced by the microbiome. However, to study microbial interactions, we need suitable computational tools that can robustly infer the microbial interaction network and subsequently disentangle it to interpret the contribution of microorganisms and their interactions with respect to their environment. This is of particular importance in medical research, where microbial interactions may be associated with the onset of certain diseases. Microbial communities, or key members thereof, are thus attractive drug targets in precision medicine [96]. Network-based approaches are powerful concepts to model and study complex relationships which can be employed in this context. Currently, the majority of network-based tools and models are used to study intra-kingdom interactions, mostly between bacteria. Most of these methods employ linear models based on correlation, regression, and probabilistic graphical models. Only few tools consider confounding factors in spite of their importance in microbiome studies. A frequently addressed bias is compositionality which results in artefactual correlations and is typically countered by pre-processing relative abundances with CLR transformation or by using a Dirichlet multinomial model. Few methods are able to distinguish direct and indirect effects using more complex conditional models and regularization. Nevertheless, simple linear models appear to be used more frequently in the literature, likely because more complex models that account for biases are plagued by a steep increase in computational complexity which leads to intolerable runtime. Moreover, network inference is hindered by a large fraction of zeros in the abundance matrices as well as by an unfavourable ratio of samples to features (curse of dimensionality). To counter these issues, relative abundances are often grouped on a higher taxonomic level, precluding insights into smaller communities. This may be mitigated by recently proposed tools such as FlashWeave, which offer bias-aware inference of microbial networks for hundreds of thousands of samples.

Species- or strain-level network analysis is not only prohibited by the additional computational complexity but also limited by the sequencing method employed. 16S rRNA amplicon sequencing method provides reliable taxonomic resolution up to genus level. In contrast, shotgun metagenomics enables species-level, and potentially strain-level resolution. Species-level network analysis may be subject to additional challenges that are currently not addressed. For instance, strain/species-level associations can be dominated by a single species as one species may comprise more than 100 different strains, yet this may not imply that all members of a particular species should be associated. This is the common phenomena observed in microbial network studies [42], [97] and is known as assortativity [98]. In other words, taxa are more likely to interact with other phylogenetically related taxa. Ha et al. [50] proposed using a standardized assortative coefficient [99] to quantify the extent of assortativity in the constructed networks of various methods but only investigated this issue for genus level and higher.

None of the existing tools successfully addresses all issues of microbial network inference. For instance, very few existing network approaches cannot reliably separate the actual ecological relationships from other pseudo (artifact) relationships. Furthermore, they typically fail to detect the nature of microbial relationships, i.e. they are unable to distinguish between competition and cooperation [21].

**Fig. 3:**
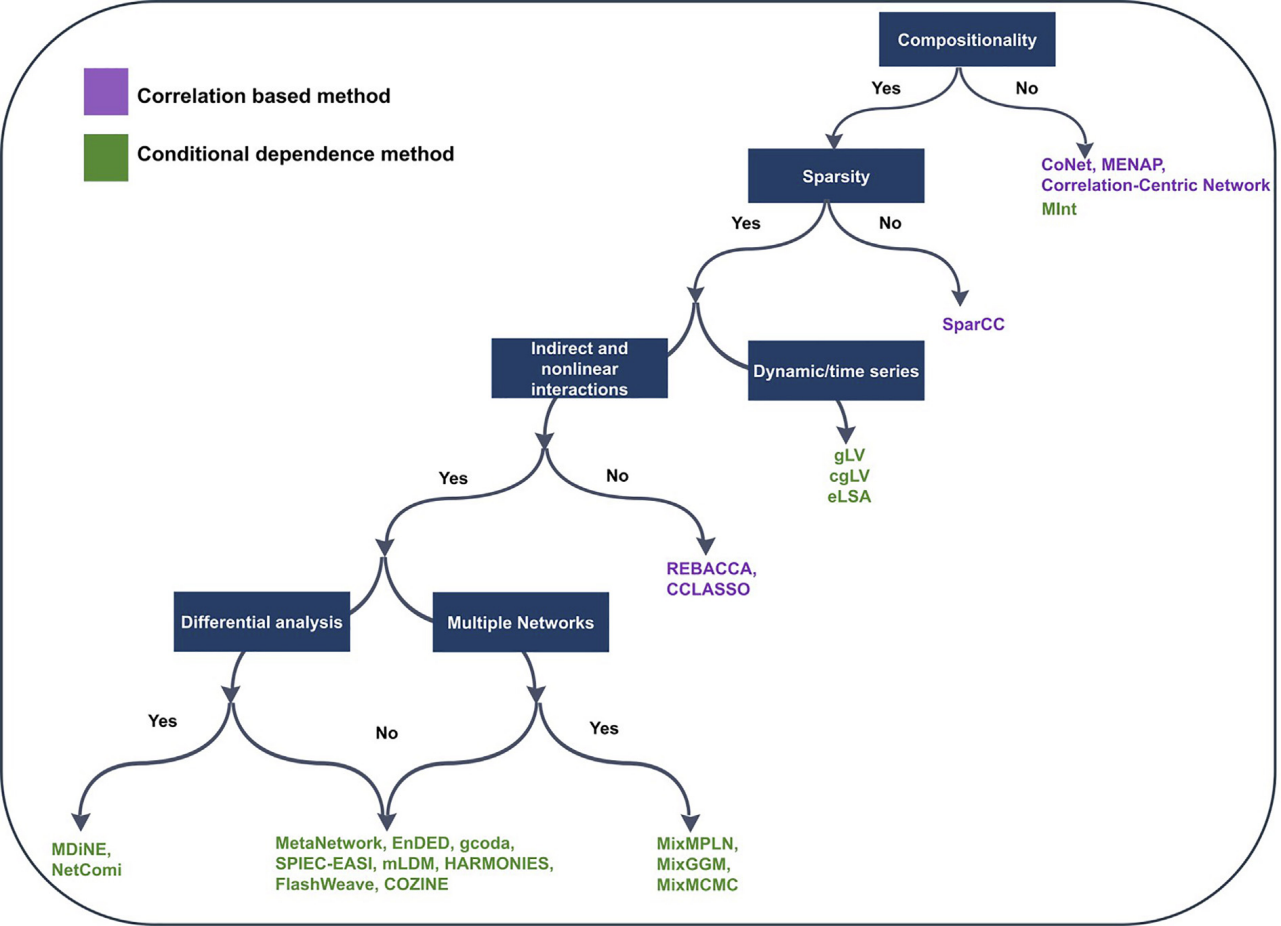
Workflow indicating the suitable network approaches depending upon different challenges.

A plethora of methods have already been developed for studying microbial interactions from a network-level perspective. Considering that many of the available tools employ similar strategies, the choice of method is a daunting task for microbiome researchers. Comprehensive guidelines are currently missing due to the lack of a suitable and comprehensive benchmark datasets or commonly accepted simulated datasets which could be used as a gold standard to systematically evaluate the performance of existing network models. Thus, users can currently choose an optimal method for their analysis only based on the trade-off between complex models that address existing biases and reveal the differences of direct and indirect relationships and faster methods which can reliably infer networks even when hundreds of taxa and thousands of samples are given. We have summarized the trade-offs in Table 1 and Fig. 3 illustrates the best options to use depending on various challenges.

Microbial interactions go far beyond within-kingdom interactions of bacteria alone. With the availability of *trans*-kingdom and multi-modal data sets including the transcriptome, metabolome and proteome, integrative network approaches are urgently needed to study *trans*-kingdom and functional interactions in the microbiome. However, very few methods have been adapted for *trans*-kingdom analysis, motivating the use of general methods developed for multi-modal integrative analysis such as MOFA+ [100]. Moreover, several data integration techniques have been used to integrate nodes from different networks, including a bipartite network [101] approach, which was used to build the community fungal-bacterial networks on the root microbiome [102] and a deep learning model allowing the integration of microbiome and metabolome [103]. Many of the existing tools currently used for inferring bacterial interaction networks have yet to be adapted for *trans*-kingdom interactions, including Gaussian graphical models, graphical lasso and mixed graphical models [104].

## 15 Conclusion

Network analysis provides valuable insights into microbial interaction networks. However, the currently available methods are not able to overcome all of the challenges associated with microbiome data including compositionality bias, overdispersion, a poor sample to feature ratio and *trans*-kingdom interactions. Analysis methods should be carefully selected based on the computational complexity that can be afforded with the data set and also with respect to the biological question that defines if it is acceptable to study the microbiome on a higher taxonomic level. In addition, further studies have to be carried out to validate these methods using universal benchmark datasets. While network analysis methods often suggest plausible hypotheses and interpretations of the data, they cannot infer causality. Integrative methods utilizing shotgun sequencing, metatranscriptomics and *meta*-metabolomics data are thus needed. Finally, efforts in computational method development need to be matched with experimental studies of microbial communities in, e.g., gnotobiology to be able to validate findings and to ultimately unravel the complexity of microbial interactions.

## CRediT authorship contribution statement

**Monica Steffi Matchado:** Writing - original draft, Visualization, Resources. **Michael Lauber:** Writing - original draft, Resources. **Sandra Reitmeier:** Writing - review & editing. **Tim Kacprowski:** Writing - review & editing. **Jan Baumbach:** Writing - review & editing. **Dirk Haller:** Writing - review & editing. **Markus List:** Conceptualization, Supervision, Project administration, Writing - original draft, Writing - review & editing.

## Declaration of Competing Interest

The authors declare that they have no known competing financial interests or personal relationships that could have appeared to influence the work reported in this paper.
