# Supplementary material for: Network analysis methods for studying microbial communities: A mini review
Source: Comput Struct Biotechnol J. 2021 May 4;19:2687–98. doi: 10.1016/j.csbj.2021.05.001 (PMC8131268; doi:10.1016/j.csbj.2021.05.001)
Supplement: Supplementary data 1 [file mmc1.docx]

**Supplementary Methods**

**Correlation based Methods**

**SparCC:** Sparse Correlations for Compositional data (SparCC) [1] is frequently used to study the microbial network, especially in human gut microbiome studies [2]–[4], as well as environmental studies [5]. SparCC iteratively estimates the linear Pearson correlations between the log transformed relative abundances. The main advantage of log ratio transformation of relative abundance is that it reflects the ratio of the true abundances present in the environment better through introducing a virtual reference point. SparCC is thus compositionally coherent, i.e., the log ratio of two OTUs is completely independent of other OTUs. Compared to direct Pearson correlations, SparCC is better suited to avoid spurious correlations [6] at the cost of higher computational complexity [7]

**CCLasso: correlation inference for compositional data through Lasso:** CCLasso is a latent variable model developed to build a regularized correlation network of microbiome data. It adapts CLR transformation to address compositionality and utilizes the least squares method with ℓ1-penalty. CCLasso performs similar to SparCC in terms of reproducibility and consistency but is better suited to avoid spurious relationships [8]. Apart from the compositionality bias, correlation methods like SparCC and CCLasso also fail to differentiate between direct and indirect edges. Indirect edges are defined as edges between two species that occur due to third factors which can be other taxa or due to environmental factors. Feizi et al. [9] and Barabási [10] developed two different methods to deal with direct and indirect effects in correlation-based methods. However, these methods are not tested against microbial co-occurrence network analysis.

**REBACCA:** Regularized Estimation of the BAsis Covariance based on Compositional dAta (REBACCA) [11] is fast composition-aware method that builds a linear, log basis covariance structure of abundances and similar to CCLasso utilizes the ℓ_1_-norm shrinkage method to compensate for indirect effects. REBACCA shows high accuracy in detecting pairwise correlations between taxa while being faster than other methods including SparCC.

**CoNet:** CoNet is a Cytoscape application inferring microbial relationships using different statistical measures including correlations (Pearson, Spearman, Kendall), similarity matrices (Steinhaus, distance correlation) and dissimilarity matrices (Euclidean, Jensen-Shannon, Kullback Leibler, Bray Curtis) [12]. The significance of the association is calculated using the ReBoot method. A special feature of CoNet is that users can assign objects to different groups such as enzymes and metabolites. By integrating this, CoNet allows not only to predict relationships within one object type but also between different object types [20]. However, the statistical measures used by CoNet to build microbial association may not be suitable for compositional data which may result in spurious correlation [12].

**Meta-Network:** Meta-Network [13] is a hybrid method combining both traditional Pearson correlation and advanced association rule mining algorithms to detect intricate (i.e. including indirect and non-linear) correlations. In contrast to correlation-based methods which capture only direct and linear relationships, Meta-Network introduces a loose definition method to retain all the interactions which are undetected by traditional methods. In the loose definition method, a co-occurrence probability matrix is calculated from a presence-absence abundance matrix. This is achieved by taking the ratio of co-existing samples to all the samples as an estimated co-occurrence probability for each pairwise species. Loose deduction method constructs the network with 80% co-occurrence probability whereas Pearson method uses 100% co-occurrence probability. Followed by this loose definition, Meta-network uses the graph-based Functional Similarity Weight (FS-Weight) algorithm to detect indirect relationships and the PCA-PMI [14] method (Path Consistency Algorithm) to infer non-linear associations. These two methods (FS-Weight and PCA-PMI) are able to independently capture many of the same nodes and edges which, according to the authors, indicates that they both can depict the complex nature of the microbial relationships. Meta-Network has further advantages, as it includes a module to perform clustering analysis and to identify key species in the co-occurrence network. It employs the MCODE algorithm [15] to identify potential clusters. Meta-Network identifies the key species by estimating the most connected and statistically significant nodes with the help of the Kruskal-Wallis test. Biological significance of these clusters and key species are assessed by using taxonomical and functional annotation databases [13].

**Correlation-Centric Network (CCN):** The majority of the microbial network tools emphasize nodes (representing taxa at different taxonomic levels) but only limited attention is given to the edges capturing their associations [16], [17]. Although these may delineate important dynamic changes of microbial co-occurrence. Correlation-Centric Network (CCN) [18] transforms the node into an edge graph where nodes represent the co-occurrence of two taxa while edges represent one of the two co-occurring taxa, respectively. First, a species-centric network (SCN) is constructed by identifying the Pearson correlation between species. Next, a binary matrix is constructed from the network graph of the SCN, where one represents the correlation between a pair of nodes, otherwise, is set to zero. This binary matrix is converted into a CCN binary matrix by calculating the correlation between the edges. Finally, the CCN network is generated from the CCN binary(incident) matrix. The concept of CCN has been utilized in other fields including biomarker discovery in gene expression studies [19]. The CCN retains all the information from the SCN and has higher connectivity than the SCN. During the network stability testing, authors showed that CCN retains stability until 65% of the nodes were removed, whereas SCNs collapsed at 35% node removal. CCNs thus provide deeper insights to detect the dynamic process of network collapse. CCNs are able to detect changes in species correlations whereas SCNs are only capable of detecting changes in species composition. Hence CCNs are recommended for longitudinal studies to predict dynamic changes in the microbial environment [18].

**MENAP:** Molecular Ecological Network Analysis Pipeline (MENAP) [20] provides a solution to choose an optimal correlation threshold which is used to filter interactions from the co-occurrence network. MENAP first builds an adjacency matrix from a count matrix using pairwise Pearson correlation coefficients. Subsequently, a random matrix theory-based method [21] is utilized to automatically identify the threshold value based on the data structure of the input to differentiate between the true correlation from noise. MENAP also features several modules to study the network organizations and to identify key species. However, MENAP does not address the issues of network sparsity and compositional bias.

**Conditional dependence Methods**

Correlation based methods and Gaussian graphical models fail to differentiate between direct and indirect associations and typically neglect the effect of confounders including biological covariates and technical errors such as sequencing depth. To account for this, a plethora of bias-aware methods have been developed which usually feature a higher computational complexity and run-time.

**MDiNE:** The aforementioned methods construct a single co-occurrence network irrespective of study conditions such as disease, treatment or control. In contrast, Microbiome Differential Network Estimation [22] generates differential networks to show how microbial relationships vary between two conditions based on an estimation of the precision matrix. MDiNE is available as an R package and addresses compositionality by utilizing a Dirichlet-multinomial logistic-normal distribution [23], [24]. Apart from handling compositionality, multinomial logistic models are also suited to handle the large number of zeros in microbial abundances without reverting to pseudo counts. MDiNE supports only a single binary covariate to construct the networks and is run-time intensive.

**Mint:** Mint (MicrobialInteraction) was developed based on Poisson-multivariate normal hierarchical model to identify the direct microbial interactions as well as to control the confounders at the multivariate normal layer, using an ℓ1 penalized precision matrix [25]. Even though it outperforms SparCC in terms of identifying the direct correlations and the effect of confounding predictors, It fails to explicitly address compositionality bias [26].

**MixMPLN:** Most of the network methods assume that the sample-taxa matrix is associated with a single network, i.e. there is only one network topology with a set of edge weights. However, a sample-taxa matrix may be derived from a larger number of biological samples where taxa may be associated with more than one network topology. Especially human gut microbiomes are associated with various factors including diet, and age and, hence, the associated microbial network may vary according to the influence of these factors. Tavakoli et al. [27] used a mixture model based on the Multivariate Poisson Log-Normal (MPLN) distribution [28] to build K microbial networks from a sample-taxa matrix associated with K underlying distributions. Following the same idea, the Mint [25] method used a Poisson-multivariate normal hierarchical model to capture the direct microbial interactions. However, the main objective of MInt is to identify direct interactions of a single network by controlling for confounders. Instead, MixMPLN constructs one network for each confounder and infers the parameters of the distributions using a maximum likelihood framework based on minorization–maximization (MM) algorithm [29]. In addition to this, MixMPLN also uses ℓ1 -penalty model to solve the sparse networks.

**NetCoMi: *Net***work *C*onstruction and comparison for ***Mi***crobiome data (NetCoMi)[30] integrates existing methods for generating single and differential microbial association networks. It implements frequently used normalization methods such as total sum scaling, cumulative sum scaling, rarefication, CLR transformation and variance stabilizing transformation for data normalization and to handle excess zeros. Various methods including SparCC, proportionality [31], SPIEC-EASI [32] and SPRING (Semi-Parametric Rank-based approach for INference in Graphical model) [33] are implemented to overcome compositionality-bias. In addition, NetCoMi features differential network analysis and differential association analysis. Differential network analysis utilizes permutation tests to evaluate the significantly different taxa between the groups. Differential association analysis uses Fisher’s z-test [34], a non-parametric resampling procedure [35] and the discordant method [36] to build differential networks that are limited to differentially associated taxa. Overall, NetCoMi gives its users considerable flexibility to select the most suitable tools or methods for a particular dataset.

**Environmentally-Driven Edge Detection (EnDED):** Ecological networks depict directed microbial interactions from a source to a target species and represent the type of interaction, e.g. competition, mutualisms or parasitism [37]. In contrast, microbial association networks are undirected and not all interactions represent true ecological relationships. EnDED [38] aims to differentiate direct and indirect associations based on environmental factors which may affect the dynamics of the ecosystem. EnDED can support both abiotic and biotic variables such as temperature, turbidity, salinity and nutrients. It employs four different approaches such as Sign Pattern [39], Overlap[38], Interaction Information[39], [40], and Data Processing Inequality [41] to identify indirect (environmentally-driven) edges. It classifies an edge as indirect due to environment factor, only if all four methods classify it as indirect.

**SPIEC-EASI:** SPIEC-EASI (SParse InversE Covariance Estimation for Ecological Association Inference) [32] is able to distinguish direct and indirect relationships in microbial network inference using the concept of conditional independence. SPIEC-EASI applies a CLR transformation to the data to address compositionality and subsequently generates a graphical network using one of two methods. The first method estimates a sparse inverse covariance matrix based on sparse graphical model inference (Glasso) [42]. All non-zero entries of the inverse covariance matrix yield the partial correlation of two taxa. The second approach, known as the Meinshausen Bühlman method, is a node wise regression model where the expression of each taxa is described by the remaining taxa [43], [44]. The local neighbourhood of a node or taxa is then described by the taxa which were used to predict its expression. Finally, the appropriate amount of sparsity of a network is inferred by using Stability Approach to Regularization Selection [45]. The final result is an undirected weighted graph where the edges imply the conditional dependency between two taxa.

**mLDM:** metagenomic Lognormal-Dirichlet-Multinomial (mLDM) is a hierarchical Bayesian model developed to capture the conditionally dependent microbial interactions and to decipher the association between micro-organisms and environmental factors by considering compositional bias. It proposes a three levels hierarchical Bayesian model. First, mLDM models the count matrix by using a multinomial distribution. Second, a Dirichlet distribution is used to model the multinomial probabilities and, finally, mLDM utilizes a multivariate log-normal distribution to model the absolute microbial abundance [46]. Overall, mLDM model is able to detect both conditionally dependent and direct associations and remove the indirect connections introduced by other common factors. mLDM performed favorably compared to Pearson and Spearman correlation, SparCC, CCLasso, CCREPE, glasso and SPIEC-EASI in terms of finding true taxa-taxa and environmental factors and taxas associations. However, this multi-layer approach leads to high computational complexity and limits both scalability and interpretability [26].

**gCoda:** Similar to Mint and mLDM, gcoda [26] was also developed to study the direct interactions of microbial community with conditional dependencies. Conditional dependence elucidates the relationships between two or more variables which are dependent on a third variable [32]. Nevertheless, these methods fail to account for compositionality and require high computational power respectively. gCoda performs CLR transformation on the relative abundance and assumes that the latent absolute abundances follow a multivariate normal distribution and that microbial interaction networks are sparse. gCoda then utilizes a logistic normal distribution and a maximum likelihood model with ℓ1 penalty to deal with sparsity. gCoda surpasses SPIEC-EASI in terms of stability, accuracy and computational complexity. Fang et al. [26] suggested that gCoda is suitable to study the microbial direct interactions with the conditional dependences.

**Hubs weighted graphical lasso:** Topological features of the network such as the node degree may introduce a bias in statistical inference, where highly connected taxa (hubs) have disproportionate influence. Methods based on regularization such as SPIEC-EASI do not consider topological network features since they implicitly assume conditional independence [47]. Recently, McGillivray [48] proposed a weighted graphical lasso approach that incorporates row/column sums as weights to penalize hub edges.

**FlashWeave:** A general issue with graphical model approaches is their lack of computational scalability. For instance, it is a common approach in methods such as SPIEC-EASI to collapse all taxa at, e.g., the family level to reduce the number of features and to achieve an acceptable run-time. Moreover, environmental and technical factors, such as the applied sequencing technology and measurement conditions, can confound analysis. To address these challenges, Tackmann et al. developed a computational approach based on a “constraint-based causal inference framework”, called FlashWeave [49]. For each taxa X, its univariate associations to all other variables are calculated and all significant results become candidates for the neighborhood of taxa X. For the final graph the neighborhood is filtered for taxas which are conditionally dependent on the taxa X given any other subset of neighbors. FlashWeave uses heuristics to achieve scalability and optionally incorporates metadata to disentangle direct microbial associations from confounding factors introduced in cross study analyses.

HARMONIES : Hybrid Approach foR MicrobiOme Network Inferences via Exploiting Sparsity (HARMONIES) [50] is a statistical framework to deal with the sparsity of microbiome data. It employs zero-inflated negative binomial distribution (ZINB) and a Dirichlet prior to deal with overdispersion and the large number of zero counts. Followed by the sample-wise normalization by ZINB, HARMONIES also utilizes the Glasso model to apply regularization in producing a sparse network. HARMONIES outperformed other methods such as Pearson, SPIEC-EASI (using both Glasso and and the MeinhausenBühlmann method), and CClasso on synthetic data, in particular when additional zeros were added.

**Network-based methods for trans-kingdom analysis**

**SPIEC-EASI Extension:** In 2018 Tripton et al. [51] adapted the SPIEC-EASI method, for trans-kingdom analysis by concatenating two or more data sets which were independently CLR-transformed. The combined data is then used to estimate a sparse inverse covariance matrix which can be interpreted as an intra- and cross-domain interaction network. Applying their method on lung and skin micro and mycobiome data, the authors showed the cross-kingdom networks had a higher overall connectivity and the modularity was reduced compared to the single-domain networks.

**Multi-Omics Factor Analysis (MOFA):** The SPIEC-EASI extension does not offer insights into underlying factors which drive the variation across samples or different groups of samples. To achieve this, Argelaguet et al. [52] proposed a method called Multi-Omics Factor Analysis (MOFA) which tries to solve this problem. proposed a method called Multi-Omics Factor Analysis (MOFA) which uses group factor analysis [53] to provide an integrative analysis of a set of samples with measurements from different data modalities. MOFA performs unsupervised matrix factorization to decompose its input matrices into a single factor matrix and a weight matrix for each of the data modalities. The factor matrix represents a small number of latent factors which capture major sources of variation across microbiome modalities and the corresponding weight matrix informs about the importance of a factor for a feature within a sample. The method supports partially overlapping samples between data modalities and can impute missing values. Downstream analysis allows to identify the most important features or taxa which drive the separation of samples and to visualize the sample in the low-dimensional space spanned by the factors. Recently, Argelaguet et al. [54] presented an extension of their model called Multi-Omics Factor Analysis v2 (MOFA+), which further accounts for side information such as experiments, batches, conditions or clinical covariates and offers improved scalability.

**Data Integration Analysis for Biomarker discovery using Latent cOmponents (DIABLO):** DIABLO [55] is a multi-omics integration tool based on partial least squares (PLS) regression, a technique to reduce the number of predictors by finding a small set of uncorrelated variables which are then used to perform least squares regression. DIABLO extracts co-expressed variables from different omics datasets to allow a discrimination between the phenotypes of interest. Applying this method on a multi omics data set consisting of microbiome, metabolome, proteome und mRNA measurements, DIABLO helped to find discriminatory biomarkers for fibromyalgia patients [56]. However, DIABLO has the assumption of a linear relationship between the selected variables which will not be satisfied in all biological systems and also the tool was developed for the use on non-categorical omics measurements of a continuous scale.

**Studying Microbiome Dynamics through** L**ocal similarity analysis**

Microbiomes tend to change their compositions in response to perturbations of their environment. Time-series analysis helps to study dynamic interaction changes in microbiomes from time series data and reveals contemporaneous patterns and factors which are responsible for changes in the community behavior.

**Local similarity analysis (LSA):** [57] is one of the widely used methods to investigate the temporal changes in microbial niche by studying interactions among the taxa as well as with the host. Alternatively, Dynamic Bayesian networks and temporal event networks can be used to study the temporal changes in microbial data. Dynamic Bayesian networks have been successfully used to study the microbial changes in infant gut microbiome [58], other longitudinal microbiome data including vaginal and oral cavity microbiome [59].

LSA analyses the time series data without replicates. However, biological experiments are typically carried out in replicates in order to estimate the variance of the measurements or to obtain a robust average [60], [61]. Extended LSA (eLSA) leverages replicates to achieve better performance in handling of missing data, and to provide confidence intervals for LSA scores [57].

**generalized Lotka–Volterra (gLV):** Lotka–Volterra models are commonly used to predict different types of interactions. While classical Lotka-Volterra models are used to predict predator-prey (competition) interaction between two species, the generalized Lotka–Volterra (gLV) uses a logistic model to simulate the growth of microbes and to infer whether an interaction of two species is competitive, amensalistic or predator-prey [62]. However, since gLV-based models estimate dynamics with respect to absolute abundance, a compositionally aware Lotka-Volterra method (cLV) [63] was developed.

**References**

[1] J. Friedman and E. J. Alm, “Inferring Correlation Networks from Genomic Survey Data,” *PLOS Comput. Biol.*, vol. 8, no. 9, p. e1002687, Sep. 2012, doi: 10.1371/journal.pcbi.1002687.

[2] Y.-N. Yu and J.-Y. Fang, “Gut Microbiota and Colorectal Cancer,” *Gastrointest. Tumors*, vol. 2, no. 1, pp. 26–32, May 2015, doi: 10.1159/000380892.

[3] A. Gorvitovskaia, S. P. Holmes, and S. M. Huse, “Interpreting Prevotella and Bacteroides as biomarkers of diet and lifestyle,” *Microbiome*, vol. 4, no. 1, p. 15, Apr. 2016, doi: 10.1186/s40168-016-0160-7.

[4] I. H. McHardy *et al.*, “Integrative analysis of the microbiome and metabolome of the human intestinal mucosal surface reveals exquisite inter-relationships,” *Microbiome*, vol. 1, no. 1, p. 17, Jun. 2013, doi: 10.1186/2049-2618-1-17.

[5] S. E. Evans and M. D. Wallenstein, “Climate change alters ecological strategies of soil bacteria,” *Ecol. Lett.*, vol. 17, no. 2, pp. 155–164, 2014, doi: 10.1111/ele.12206.

[6] S. Weiss *et al.*, “Correlation detection strategies in microbial data sets vary widely in sensitivity and precision,” *ISME J.*, vol. 10, no. 7, pp. 1669–1681, Jul. 2016, doi: 10.1038/ismej.2015.235.

[7] H. Hirano and K. Takemoto, “Difficulty in inferring microbial community structure based on co-occurrence network approaches,” *BMC Bioinformatics*, vol. 20, no. 1, p. 329, Jun. 2019, doi: 10.1186/s12859-019-2915-1.

[8] H. Fang, C. Huang, H. Zhao, and M. Deng, “CCLasso: correlation inference for compositional data through Lasso,” *Bioinformatics*, vol. 31, no. 19, pp. 3172–3180, Oct. 2015, doi: 10.1093/bioinformatics/btv349.

[9] S. Feizi, D. Marbach, M. Médard, and M. Kellis, “Network deconvolution as a general method to distinguish direct dependencies in networks,” *Nat. Biotechnol.*, vol. 31, no. 8, p. 726, Aug. 2013, doi: 10.1038/nbt.2635.

[10] B. Barzel and A.-L. Barabási, “Network link prediction by global silencing of indirect correlations,” *Nat. Biotechnol.*, vol. 31, no. 8, pp. 720–725, Aug. 2013, doi: 10.1038/nbt.2601.

[11] Y. Ban, L. An, and H. Jiang, “Investigating microbial co-occurrence patterns based on metagenomic compositional data,” *Bioinforma. Oxf. Engl.*, vol. 31, no. 20, pp. 3322–3329, Oct. 2015, doi: 10.1093/bioinformatics/btv364.

[12] K. Faust and J. Raes, “CoNet app: inference of biological association networks using Cytoscape,” *F1000Research*, vol. 5, Oct. 2016, doi: 10.12688/f1000research.9050.2.

[13] P. Yang, S. Yu, L. Cheng, and K. Ning, “Meta-network: optimized species-species network analysis for microbial communities,” *BMC Genomics*, vol. 20, no. 2, p. 187, Apr. 2019, doi: 10.1186/s12864-019-5471-1.

[14] S. Marino, N. T. Baxter, G. B. Huffnagle, J. F. Petrosino, and P. D. Schloss, “Mathematical modeling of primary succession of murine intestinal microbiota,” *Proc. Natl. Acad. Sci.*, vol. 111, no. 1, pp. 439–444, Jan. 2014, doi: 10.1073/pnas.1311322111.

[15] J. Wang, J. Zhong, G. Chen, M. Li, F. Wu, and Y. Pan, “ClusterViz: A Cytoscape APP for Cluster Analysis of Biological Network,” *IEEE/ACM Trans. Comput. Biol. Bioinform.*, vol. 12, no. 4, pp. 815–822, Aug. 2015, doi: 10.1109/TCBB.2014.2361348.

[16] J. K. Huang *et al.*, “Systematic Evaluation of Molecular Networks for Discovery of Disease Genes,” *Cell Syst.*, vol. 6, no. 4, pp. 484-495.e5, Apr. 2018, doi: 10.1016/j.cels.2018.03.001.

[17] R. De Smet and K. Marchal, “Advantages and limitations of current network inference methods,” *Nat. Rev. Microbiol.*, vol. 8, no. 10, pp. 717–729, Oct. 2010, doi: 10.1038/nrmicro2419.

[18] P. Yang, C. Tan, M. Han, L. Cheng, X. Cui, and K. Ning, “Correlation-Centric Network (CCN) representation for microbial co-occurrence patterns: new insights for microbial ecology,” *NAR Genomics Bioinforma.*, vol. 2, no. 2, Jun. 2020, doi: 10.1093/nargab/lqaa042.

[19] X. Yu, G. Li, and L. Chen, “Prediction and early diagnosis of complex diseases by edge-network,” *Bioinforma. Oxf. Engl.*, vol. 30, no. 6, pp. 852–859, Mar. 2014, doi: 10.1093/bioinformatics/btt620.

[20] Y. Deng, Y.-H. Jiang, Y. Yang, Z. He, F. Luo, and J. Zhou, “Molecular ecological network analyses,” *BMC Bioinformatics*, vol. 13, no. 1, p. 113, May 2012, doi: 10.1186/1471-2105-13-113.

[21] F. Luo *et al.*, “Constructing gene co-expression networks and predicting functions of unknown genes by random matrix theory,” *BMC Bioinformatics*, vol. 8, no. 1, p. 299, Aug. 2007, doi: 10.1186/1471-2105-8-299.

[22] K. McGregor, A. Labbe, and C. M. T. Greenwood, “MDiNE: a model to estimate differential co-occurrence networks in microbiome studies,” *Bioinformatics*, vol. 36, no. 6, pp. 1840–1847, Mar. 2020, doi: 10.1093/bioinformatics/btz824.

[23] I. Holmes, K. Harris, and C. Quince, “Dirichlet Multinomial Mixtures: Generative Models for Microbial Metagenomics,” *PLoS ONE*, vol. 7, no. 2, Feb. 2012, doi: 10.1371/journal.pone.0030126.

[24] J. Chen and H. Li, “Variable selection for sparse Dirichlet-multinomial regression with an application to microbiome data analysis,” *Ann. Appl. Stat.*, vol. 7, no. 1, pp. 418–442, Mar. 2013, doi: 10.1214/12-AOAS592.

[25] S. Biswas, M. Mcdonald, D. S. Lundberg, J. L. Dangl, and V. Jojic, “Learning Microbial Interaction Networks from Metagenomic Count Data,” *J. Comput. Biol. J. Comput. Mol. Cell Biol.*, vol. 23, no. 6, pp. 526–535, Jun. 2016, doi: 10.1089/cmb.2016.0061.

[26] H. Fang, C. Huang, H. Zhao, and M. Deng, “gCoda: Conditional Dependence Network Inference for Compositional Data,” *J. Comput. Biol.*, vol. 24, no. 7, pp. 699–708, Jul. 2017, doi: 10.1089/cmb.2017.0054.

[27] S. Tavakoli and S. Yooseph, “Learning a mixture of microbial networks using minorization–maximization,” *Bioinformatics*, vol. 35, no. 14, pp. i23–i30, Jul. 2019, doi: 10.1093/bioinformatics/btz370.

[28] J. Aitchison and C. H. Ho, “The multivariate Poisson-log normal distribution,” *Biometrika*, vol. 76, no. 4, pp. 643–653, Dec. 1989, doi: 10.1093/biomet/76.4.643.

[29] H. Zhou and K. Lange, “MM Algorithms for Some Discrete Multivariate Distributions,” *J. Comput. Graph. Stat. Jt. Publ. Am. Stat. Assoc. Inst. Math. Stat. Interface Found. N. Am.*, vol. 19, no. 3, pp. 645–665, Sep. 2010, doi: 10.1198/jcgs.2010.09014.

[30] S. Peschel, C. L. Müller, E. von Mutius, A.-L. Boulesteix, and M. Depner, “NetCoMi: network construction and comparison for microbiome data in R,” *Brief. Bioinform.*, no. bbaa290, Dec. 2020, doi: 10.1093/bib/bbaa290.

[31] D. Lovell, V. Pawlowsky-Glahn, J. J. Egozcue, S. Marguerat, and J. Bähler, “Proportionality: a valid alternative to correlation for relative data,” *PLoS Comput. Biol.*, vol. 11, no. 3, p. e1004075, Mar. 2015, doi: 10.1371/journal.pcbi.1004075.

[32] Z. D. Kurtz, C. L. Müller, E. R. Miraldi, D. R. Littman, M. J. Blaser, and R. A. Bonneau, “Sparse and Compositionally Robust Inference of Microbial Ecological Networks,” *PLOS Comput. Biol.*, vol. 11, no. 5, p. e1004226, May 2015, doi: 10.1371/journal.pcbi.1004226.

[33] G. Yoon, I. Gaynanova, and C. L. Müller, “Microbial Networks in SPRING - Semi-parametric Rank-Based Correlation and Partial Correlation Estimation for Quantitative Microbiome Data,” *Front. Genet.*, vol. 10, 2019, doi: 10.3389/fgene.2019.00516.

[34] R. A. Fisher, “Statistical Methods for Research Workers,” in *Breakthroughs in Statistics: Methodology and Distribution*, S. Kotz and N. L. Johnson, Eds. New York, NY: Springer, 1992, pp. 66–70.

[35] R. Gill, S. Datta, and S. Datta, “A statistical framework for differential network analysis from microarray data,” *BMC Bioinformatics*, vol. 11, p. 95, Feb. 2010, doi: 10.1186/1471-2105-11-95.

[36] C. Siska, R. Bowler, and K. Kechris, “The discordant method: a novel approach for differential correlation,” *Bioinform.*, 2016, doi: 10.1093/bioinformatics/btv633.

[37] Y. Xiao, M. T. Angulo, J. Friedman, M. K. Waldor, S. T. Weiss, and Y.-Y. Liu, “Mapping the ecological networks of microbial communities,” *Nat. Commun.*, vol. 8, no. 1, p. 2042, 11 2017, doi: 10.1038/s41467-017-02090-2.

[38] I. M. Deutschmann *et al.*, “Disentangling environmental effects in microbial association networks,” In Review, preprint, Aug. 2020. doi: 10.21203/rs.3.rs-57387/v1.

[39] G. Lima-Mendez *et al.*, “Determinants of community structure in the global plankton interactome,” *Science*, vol. 348, no. 6237, pp. 1262073–1262073, May 2015, doi: 10.1126/science.1262073.

[40] A. Ghassami and N. Kiyavash, “Interaction information for causal inference: The case of directed triangle,” in *2017 IEEE International Symposium on Information Theory (ISIT)*, Jun. 2017, pp. 1326–1330, doi: 10.1109/ISIT.2017.8006744.

[41] Thomas M. Cover and Joy A. Thomas, “Inequalities in Information Theory,” in *Elements of Information Theory*, John Wiley & Sons, Ltd, 2001, pp. 482–509.

[42] J. Friedman, T. Hastie, and R. Tibshirani, “Sparse inverse covariance estimation with the graphical lasso,” *Biostatistics*, vol. 9, no. 3, pp. 432–441, Jul. 2008, doi: 10.1093/biostatistics/kxm045.

[43] N. Meinshausen and P. Bühlmann, “High-dimensional graphs and variable selection with the Lasso,” *Ann. Stat.*, vol. 34, no. 3, pp. 1436–1462, Jun. 2006, doi: 10.1214/009053606000000281.

[44] R. Tibshirani, “Regression Shrinkage and Selection via the Lasso,” *J. R. Stat. Soc. Ser. B Methodol.*, vol. 58, no. 1, pp. 267–288, 1996.

[45] H. Liu, K. Roeder, and L. Wasserman, “Stability Approach to Regularization Selection (StARS) for High Dimensional Graphical Models,” *Adv. Neural Inf. Process. Syst.*, vol. 24, no. 2, pp. 1432–1440, Dec. 2010.

[46] Y. Yang, N. Chen, and T. Chen, “Inference of Environmental Factor-Microbe and Microbe-Microbe Associations from Metagenomic Data Using a Hierarchical Bayesian Statistical Model,” *Cell Syst.*, vol. 4, no. 1, pp. 129-137.e5, Jan. 2017, doi: 10.1016/j.cels.2016.12.012.

[47] K. M. Tan, P. London, K. Mohan, S.-I. Lee, M. Fazel, and D. Witten, “Learning Graphical Models With Hubs,” *J. Mach. Learn. Res. JMLR*, vol. 15, pp. 3297–3331, Oct. 2014.

[48] A. McGillivray, A. Khalili, and D. A. Stephens, “Estimating sparse networks with hubs,” *J. Multivar. Anal.*, vol. 179, p. 104655, Sep. 2020, doi: 10.1016/j.jmva.2020.104655.

[49] J. Tackmann, J. F. Matias Rodrigues, and C. von Mering, “Rapid Inference of Direct Interactions in Large-Scale Ecological Networks from Heterogeneous Microbial Sequencing Data,” *Cell Syst.*, vol. 9, no. 3, pp. 286-296.e8, Sep. 2019, doi: 10.1016/j.cels.2019.08.002.

[50] S. Jiang *et al.*, “HARMONIES: A Hybrid Approach for Microbiome Networks Inference via Exploiting Sparsity,” *Front. Genet.*, vol. 11, Jun. 2020, doi: 10.3389/fgene.2020.00445.

[51] L. Tipton *et al.*, “Fungi stabilize connectivity in the lung and skin microbial ecosystems,” *Microbiome*, vol. 6, no. 1, p. 12, Jan. 2018, doi: 10.1186/s40168-017-0393-0.

[52] R. Argelaguet *et al.*, “Multi-Omics Factor Analysis—a framework for unsupervised integration of multi-omics data sets,” *Mol. Syst. Biol.*, vol. 14, no. 6, p. e8124, Jun. 2018, doi: 10.15252/msb.20178124.

[53] S. Virtanen, A. Klami, S. Khan, and S. Kaski, “Bayesian Group Factor Analysis,” in *Proceedings of the Fifteenth International Conference on Artificial Intelligence and Statistics*, La Palma, Canary Islands, Apr. 2012, vol. 22, pp. 1269–1277, [Online]. Available: http://proceedings.mlr.press/v22/virtanen12.html.

[54] R. Argelaguet *et al.*, “MOFA+: a statistical framework for comprehensive integration of multi-modal single-cell data,” *Genome Biol.*, vol. 21, no. 1, p. 111, May 2020, doi: 10.1186/s13059-020-02015-1.

[55] A. Singh *et al.*, “DIABLO: an integrative approach for identifying key molecular drivers from multi-omics assays,” *Bioinformatics*, vol. 35, no. 17, pp. 3055–3062, Sep. 2019, doi: 10.1093/bioinformatics/bty1054.

[56] M. Clos-Garcia *et al.*, “Gut microbiome and serum metabolome analyses identify molecular biomarkers and altered glutamate metabolism in fibromyalgia,” *EBioMedicine*, vol. 46, pp. 499–511, Aug. 2019, doi: 10.1016/j.ebiom.2019.07.031.

[57] Q. Ruan, D. Dutta, M. S. Schwalbach, J. A. Steele, J. A. Fuhrman, and F. Sun, “Local similarity analysis reveals unique associations among marine bacterioplankton species and environmental factors,” *Bioinforma. Oxf. Engl.*, vol. 22, no. 20, pp. 2532–2538, Oct. 2006, doi: 10.1093/bioinformatics/btl417.

[58] M. J. McGeachie *et al.*, “Longitudinal Prediction of the Infant Gut Microbiome with Dynamic Bayesian Networks,” *Sci. Rep.*, vol. 6, no. 1, Art. no. 1, Feb. 2016, doi: 10.1038/srep20359.

[59] J. Lugo-Martinez, D. Ruiz-Perez, G. Narasimhan, and Z. Bar-Joseph, “Dynamic interaction network inference from longitudinal microbiome data,” *Microbiome*, vol. 7, no. 1, p. 54, Apr. 2019, doi: 10.1186/s40168-019-0660-3.

[60] M. L. Lee, F. C. Kuo, G. A. Whitmore, and J. Sklar, “Importance of replication in microarray gene expression studies: statistical methods and evidence from repetitive cDNA hybridizations,” *Proc. Natl. Acad. Sci. U. S. A.*, vol. 97, no. 18, pp. 9834–9839, Aug. 2000, doi: 10.1073/pnas.97.18.9834.

[61] T. T. Nguyen, R. R. Almon, D. C. DuBois, W. J. Jusko, and I. P. Androulakis, “Importance of replication in analyzing time-series gene expression data: Corticosteroid dynamics and circadian patterns in rat liver,” *BMC Bioinformatics*, vol. 11, no. 1, p. 279, May 2010, doi: 10.1186/1471-2105-11-279.

[62] C. Li, K. R. Chng, J. S. Kwah, T. V. Av-Shalom, L. Tucker-Kellogg, and N. Nagarajan, “An expectation-maximization algorithm enables accurate ecological modeling using longitudinal microbiome sequencing data,” *Microbiome*, vol. 7, no. 1, p. 118, Aug. 2019, doi: 10.1186/s40168-019-0729-z.

[63] T. A. Joseph, L. Shenhav, J. B. Xavier, E. Halperin, and I. Pe’er, “Compositional Lotka-Volterra describes microbial dynamics in the simplex,” *PLOS Comput. Biol.*, vol. 16, no. 5, p. e1007917, May 2020, doi: 10.1371/journal.pcbi.1007917.
